# Multiscale Entropy Algorithms to Analyze Complexity and Variability of Trunk Accelerations Time Series in Subjects with Parkinson’s Disease

**DOI:** 10.3390/s23104983

**Published:** 2023-05-22

**Authors:** Stefano Filippo Castiglia, Dante Trabassi, Carmela Conte, Alberto Ranavolo, Gianluca Coppola, Gabriele Sebastianelli, Chiara Abagnale, Francesca Barone, Federico Bighiani, Roberto De Icco, Cristina Tassorelli, Mariano Serrao

**Affiliations:** 1 Department of Medical and Surgical Sciences and Biotechnologies, “Sapienza” University of Rome, Polo Pontino, 04100 Latina, Italy; stefanofilippo.castiglia@uniroma1.it (S.F.C.); carmelaconte536@gmail.com (C.C.); gianluca.coppola@uniroma1.it (G.C.); gabriele.sebastianelli@uniroma1.it (G.S.); chiara.abagnale@uniroma1.it (C.A.); francesca.barone@uniroma1.it (F.B.); mariano.serrao@uniroma1.it (M.S.); 2 Department of Occupational and Environmental Medicine, Epidemiology and Hygiene, INAIL, 00078 Monte Porzio Catone, Italy; 3 Department of Brain and Behavioral Sciences, University of Pavia, 27100 Pavia, Italy; a.ranavolo@inail.it (A.R.); federico.bighiani@mondino.it (F.B.); roberto.deicco@mondino.it (R.D.I.); cristina.tassorelli@unipv.it (C.T.); 4 Movement Analysis Research Unit, IRCSS Mondino Foundation, 27100 Pavia, Italy; 5 Movement Analysis Laboratory, Policlinico Italia, 00162 Rome, Italy

**Keywords:** multiscale sample entropy, refine composite multiscale entropy, cerebellar ataxia, Parkinson’s disease, trunk acceleration time series, complexity index, gait variability, gait complexity, gait pattern, movement disorders

## Abstract

The aim of this study was to assess the ability of multiscale sample entropy (MSE), refined composite multiscale entropy (RCMSE), and complexity index (CI) to characterize gait complexity through trunk acceleration patterns in subjects with Parkinson’s disease (swPD) and healthy subjects, regardless of age or gait speed. The trunk acceleration patterns of 51 swPD and 50 healthy subjects (HS) were acquired using a lumbar-mounted magneto-inertial measurement unit during their walking. MSE, RCMSE, and CI were calculated on 2000 data points, using scale factors (*τ*) 1–6. Differences between swPD and HS were calculated at each *τ*, and the area under the receiver operating characteristics, optimal cutoff points, post-test probabilities, and diagnostic odds ratios were calculated. MSE, RCMSE, and CIs showed to differentiate swPD from HS. MSE in the anteroposterior direction at *τ*4 and *τ*5, and MSE in the ML direction at *τ*4 showed to characterize the gait disorders of swPD with the best trade-off between positive and negative posttest probabilities and correlated with the motor disability, pelvic kinematics, and stance phase. Using a time series of 2000 data points, a scale factor of 4 or 5 in the MSE procedure can yield the best trade-off in terms of post-test probabilities when compared to other scale factors for detecting gait variability and complexity in swPD.

## 1. Introduction

Subjects with Parkinson’s disease (swPD) experience progressively invalidating gait impairment [1], which affects their quality of life and increases their risk of falling [1,2,3,4].

Due to the effects of dopamine depletion on motor control, swPD are characterized by increased gait variability [5,6,7], which can result in a number of gait abnormalities, including shuffling gait and reduced step length [8,9,10]. Altered trunk behavior showed to characterize gait impairment [11,12,13,14,15,16,17] and to represent a responsive outcome for medications and rehabilitation in swPD [17,18,19,20,21,22]. Wearable sensors, such as magneto-inertial measurement units (MIMUs), have been shown to provide trunk acceleration-derived gait indexes that can accurately characterize gait abnormalities, fall risk, and gait variability in swPD [14,23,24], and responsive measures to quantify the effectiveness of rehabilitation [25]. 

When retrieved from trunk accelerations, the coefficient of variation (CV), a commonly used statistical measure that quantifies the variability of spatio-temporal gait parameters [13,26,27,28,29], can be extrapolated. However, CV may present some limitations in assessing gait variabilities in swPD, such as high dependency on gait speed, limited ability to provide information on the underlying patterns and short-term changes in gait variability [9,13,30], and lack of identification of gait variability at earlier stages of the disease [31]. Moreover, CV is dependent on the identification of gait cycles, which is a possible source of error due to irregular acceleration signals or difficulties in the identification of acceleration peaks, particularly in neurodegenerative diseases [32,33,34,35]. 

To overcome these shortcomings, researchers have proposed adopting nonlinear entropy measures, which assess gait variability by providing a measure of the complexity and regularity of a time series, regardless of step detection [36,37,38,39]. 

Entropy quantifies the probability of the next state of the system based on what is known about the current state of a time series [40,41]. When the probability is high, the following system states provide little new information, resulting in low entropy values. When the probability is low, the subsequent data points in the system provide a greater amount of new information, resulting in high entropy values, indicating greater gait irregularity or complexity of the gait pattern. Several methods for calculating entropy have been proposed [41,42,43,44,45,46]. Among them, sample entropy (SampEn) [43]-based methods have been described as valid tools for assessing gait regularity in healthy subjects and pathological conditions [40,47,48], including trunk acceleration-derived gait signals from swPD [49]. Multiscale entropy (MSE) and refined composite multiscale entropy (RCMSE) have been shown to be the most appropriate entropy measures for assessing the repeatability of gait signals, particularly when analyzing shorter time series [41,44], such as those generated by ambulatory gait trials, where they limit the risk of noisy and unstable entropy estimates [43,50,51,52].

MSE is an extension of SampEn that computes SampEn at different scales by segmenting the original time series into different length windows through a coarse-graining procedure [53,54,55,56,57,58]. When MSE was applied to trunk accelerations, it revealed differences between treadmill and overground walking in older but not younger individuals [59], and a progressive decrease in trunk acceleration complexity from childhood to adulthood during natural walking [60]. RCMSE has been proposed to overcome the probability of undefined entropy of MSE [61] by calculating the entropies of each coarse-grained time series into a composite multiscale algorithm with a scale factor [61]. Recently, another method of entropy calculations, the complexity index (CI), has been introduced to assess the gait complexity of swPD across a pre-determined range of scale factors [62,63,64]. However, its ability to characterize the gait of swPD, compared to healthy subjects, has never been investigated.

Notably, when calculating MSE and RCMSE, researchers should consider which combination of signal embedding, tolerance radius, scale factor, and length of data best fit with their type of data and study objectives [40]. The length of 2000 data points (*N*) was described as an acceptable compromise between instability of the results and loss of significant information, a signal embedding value (*m*) of 2 was calculated using the nearest neighbor method [65,66,67] and 0.2 times the standard deviation was used as the tolerance radius (*r*). However, the choice of number of scales, commonly referred to as *τ*, differs across the studies analyzing MSE and RCMSE [40,41]. As it may significantly affect sample entropy calculations, the optimal *τ* related to *N* to identify complexity and irregularity should be identified for each pathological condition [38,68]. 

Furthermore, when assessing the discriminative ability of entropy measures, the effects of age [37] and gait speed [69], which can overrepresent the differences between pathological and healthy gait [70], should be considered. 

Therefore, the aims of this study were: (i) to identify the best *τ* in MSE or RCMSE procedure, or the ability of CI, to characterize the complexity and variability of trunk acceleration patterns of swPD during gait, compared with healthy subjects (HS), regardless of age and gait speed; (ii) to assess the ability of MSE and RCMSE calculated using the identified optimal *τ,* and CI to characterize fallers within swPD; (iii) to assess the ability of MSE and RCMSE as calculated through the identified optimal *τ*, and CI to differentiate swPD according to their disability stages; and (iv) identify correlations between MSE and RCMSE at the optimal *τ*, and CI, with clinical features and spatio-temporal and kinematic gait parameters in swPD. 

We hypothesized that MSE and/or RCMSE at a single *τ*, or CI, could characterize trunk irregularity in swPD, regardless of age and gait speed, and that could reflect the clinical status and kinematic gait abnormalities.

## 2. Materials and Methods

### 2.1. Subjects

Gait data from 51 swPD, acquired at “ICOT”, Latina, Italy, and at “IRCSS Casimiro Mondino”, Pavia Italy, were included in the study. SwPD were included according to the following inclusion criteria: (i) idiopathic PD diagnosis based on UK bank criteria [71]; (ii) Hoehn and Yahr (HY) scale classification 1–3 [72]; (iii) ability to walk unassisted for at least 30 m along a laboratory corridor without presenting freezing of gait; and (iv) a stable and accustomed drug dosage for at least 2 weeks prior to the gait assessment. Subjects with cognitive deficits as defined by Mini-Mental State Examination score <26 [73,74], moderate-to-severe depression, as defined by Back Depression Inventory scores >17 [75,76], orthopedic or other diseases influencing gait behavior, such as other neurological conditions, clinically defined osteoarthritis, joint replacements, and subjects reporting hip or knee joint pain, limited hip range of motion, or anatomic alterations of the joints, were excluded [77,78,79]. Gait data from 50 age and gait speed-matched healthy subjects (HS) were included for comparison. To match pwPD and HS, a 1:1 optimal matching procedure using the propensity score difference method was conducted [80]. Each HS was asked to repeat the gait task twice while walking at both their self-selected speed and a slower directed speed in order to reduce the effect of gait speed on the other speed-dependent gait parameters and to gather the largest sample size for speed-matched comparisons [17,81]. Age and gait speed were used as covariates in logistic regression analysis to calculate the propensity scores [82,83,84]. Table 1 summarizes the clinical characteristics of the included subjects. All participants gave their informed consent in accordance with the Helsinki Declaration, and the study was approved by the local ethics committee (CE Lazio2 protocol n.° 0053667/2021).

### 2.2. Procedures

**Figure 1:**
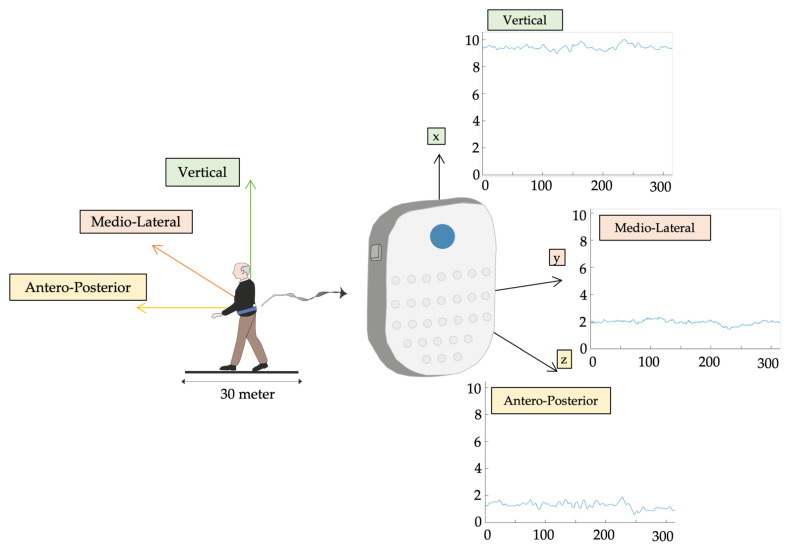
Triaxial trunk acceleration. Triaxial trunk acceleration extrapolated by L5 inertial sensor after subjects were asked to walk barefoot down a 30-m-long corridor at a self-selected speed.

Data were collected using an inertial sensor (BTS GWALK, BTS, Milan, Italy) positioned at L5 via a unique ergonomic belt. The “Walk+” protocol of the G-STUDIO software vers.3.5.25.0 (BTS, Milan, Italy) was used to detect the linear acceleration patterns of the trunk during gait in the anterior-posterior (AP), medio-lateral (ML), and vertical (V) directions, and spatio-temporal parameters and pelvic kinematics, at a sampling rate of 100Hz. A triaxial accelerometer and gyroscope (16 bit/axis) and a triaxial magnetometer are included in the sensor (13 bit). Spatio-temporal characteristics of the included sample are described in Table 1. As sample entropy algorithms are sensitive to concatenation of gait trials [85], to collect the largest number of consecutive data points, subjects were asked to walk through a 30 m long pathway at their own pace (Figure 1). 

As this study focused on natural locomotion, participants were allowed to choose their desired speed without interfering with their rhythm or receiving external sensory information. HS were also requested to walk at a slower pace to increase the sample size for the matching procedure. The multiscale entropy techniques were calculated using the MATLAB software (MATLAB R2022a 7.4.0, MathWorks, Natick, MA, USA). 

### 2.3. Entropy Algorithms

To calculate the entropy measures, we chose an embedding dimension, *m* = 2 [86], and a fixed tolerance, *r* = 0.2, multiplied by the standard deviation [87]. Due to the amount of information shared from point-to-point decreases as the lag increases, we chose a standard time lag = 1 [88]. A scale factor τ = 1–6 was chosen as the most appropriate scale factor used in previous gait research work, based on the number of data points evaluated (*N* = 2000 [41]).

#### 2.3.1. Multiscale Entropy (MSE)

**Figure 2:**
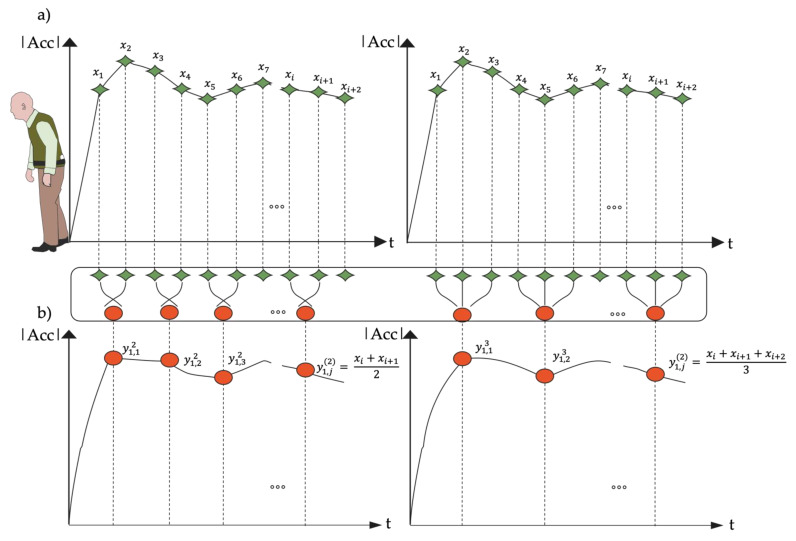
Coarse Graining Procedure. Given a time series $x_{1},\;x_{2}\ldots x_{i+2}$, we first create coarse-grained time series by averaging a growing number of data points in non-overlapping windows. Schematic illustration of the coarse-graining procedure in Multiscale Sample Entropy for scale *τ* = 2 in (**a**) and for scale *τ* = 3 in (**b**); data length of the trunk acceleration time series reduced, respectively to $\frac{N}{2}\;$ and $\frac{N}{3}$.

The *MSE* calculation consisted of two procedures: (i) a coarse-graining procedure for obtaining representations of the original time series on various time scales (Figure 2); and (ii) the *SampEn* procedure for quantifying the coarse-grained time series’ regularities [89]. The original time series was separated into non-overlapping windows of length, and the data points inside each window were averaged to generate the coarse-grained time series at a scale factor of *τ*. As illustrated in Figure 2, coarse-grained time series are separated by a scale factor of *τ* for the original time series.

*SampEn* was calculated as follows: 

Let *x* = {$x_{1},\;x_{2}\ldots x_{N}$} represent a time series of length *N*. 

Using Equation (1), build model vectors of size *m*:

(1) $$x_{i}^{m}=\left\{x_{i}\;\;x_{i+1}\;\ldots \;x_{i+m-1}\right\},\;1\leq i\leq N-m\;$$

**Figure 3:**
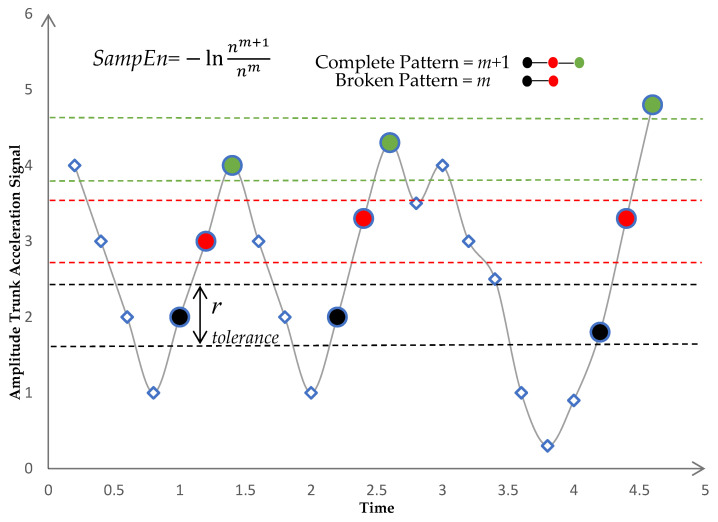
Sample Entropy calculation. For each pattern of m points in trunk acceleration signal, places in other parts of the signal where the template is seen are identified within tolerance r. Sample Entropy is calculated as the negative natural of the conditional probability that the pattern of m + 1 points will match if that the pattern of m points did match. After the signal matched the first two parts of the pattern m, this is the probability that pattern match will complete, m + 1. The number of m matches are compared to the number of complete pattern (m + 1) matches.

1. There will be correspondence if the distance between two vectors (x*^m^_i_*, x*^m^_j_*) is smaller than a predefined tolerance *r*. The distance between the two vectors was calculated using the norm of infinity:
  (2) $$d_{ij}^{m}=∥x_{i}^{m}-x_{j}^{m}∥{}_{\infty },\;1\leq i,\;j\leq N-m,\;j\neq i\;$$
2. If $d_{ij}^{m}$ was less than or equal to the predefined tolerance r, we defined (x*^m^_i_*, x*^m^_j_*) a pair of *m*-dimensional matched vectors. Total number of pairs of *m*-dimensional matched vectors, given *nm*.
3. We repeated steps 1–3 for *m* = *m* + 1, where *n^m^*^+1^ represents the total number of (*m* + 1) dimensional matched vector pairs as shown in Figure 3.
4. The *SampEn* was defined as the logarithm of the ratio of $n^{m+1}$ to $n^{m}$ as in Equation (3):
  (3) $$SampEn\left(x,\;m,r\right)=-\ln\frac{n^{m+1}}{n^{m}}$$

The *k*-th coarse-grained time series $y_{k}^{\left(\tau \right)}=\left\{y_{k,1}^{\left(\tau \right)}\;y_{k,2}^{\left(\tau \right)}\ldots \;y_{k,p}^{\left(\tau \right)}\right\}$ of *x* was defined as follows:

(4) $$y_{k,j}^{\left(\tau \right)}=\frac{1}{\tau }\;\sum_{i=\left(j-1\right)\tau +k}^{j\tau +k-1}x_{i},\;1\leq j\leq \frac{N}{\tau },\;1\leq k\leq \tau$$

As in the conventional MSE algorithm proposed by Costa et al. [44], the MSE at a scale factor of *τ* was defined as the *SampEn* of the first coarse-grained time series as in Equation (5):

(5) $$MSE\left(x,\;\tau ,\;m,r\right)=SampEn\left(y_{1}^{\left(\tau \right)},\;m,r\;\right)\;$$

#### 2.3.2. Refined Composite Multiscale Entropy (RCMSE)

To calculate *RCMSE*, the SampEns of all coarse-grained time series were calculated in the *CMSE* algorithm at a scale factor of *τ*, and the *CMSE* value was defined as the mean of *τ* SampEns:

(6) $$CMSE\left(x,\tau ,m,r\right)=\frac{1}{\tau }\overset{\tau }{\underset{k=1}{\sum }}SampEn\left(y_{k}^{\left(\tau \right)},m,r\right)=\frac{1}{\tau }\overset{\tau }{\underset{k=1}{\sum }}\left(-ln\frac{n_{k,\tau }^{m+1}}{n_{k,\tau }^{m}}\right)$$

where $n_{k,\tau }^{m}$ represents the total number of m-dimensional matched vector pairs and is constructed from the *k*-*th* coarse grained time series at a scale factor of τ. 

The logarithms of the ratio of $n_{k,\tau }^{m+1}$ to $n_{k,\tau }^{m}$ for all τ coarse-grained series are investigated first in the *CMSE* algorithm, and the average of these logarithms is then determined as the entropy value. When one of the values of $n_{k,\tau }^{m+1}$ to $n_{k,\tau }^{m}$ is 0, the *CMSE* value is undefined. The likelihood of inducing undefined entropy is higher when the *CMSE* is used to examine a short time series than when the *MSE* is used. Due to this flaw, the *CMSE* algorithm’s short time series analysis applications are limited. To overcome this issue, Wu et al. introduced the *RCMSE* method [61]. The *RCMSE* algorithm was calculated according to following steps:

(1) To obtain coarse-grained time series on different time scales, we utilized the coarse-graining process indicated in Equation (4).
(2) For all τ coarse-grained series, the number of matched vector pairs, $n_{k,\tau }^{m+1}$ and $n_{k,\tau }^{m}$, was determined at a scale factor of τ.
(3) For 1≤k≤τ, let ${\bar{n}}_{k,\tau }^{m}\;\left({\bar{n}}_{k,\tau }^{m+1}\right)$ denote the mean of $n_{k,\tau }^{m}\;\left(n_{k,\tau }^{m+1}\right)$. Equation (7) provides the *RCMSE* value at a scale factor of τ.
  (7) $$RCMSE\left(x,\tau ,m,r\right)=-\ln\left(\frac{{\bar{n}}_{k,\tau }^{m+1}}{{\bar{n}}_{k,\tau }^{m}}\right)$$
  where ${\bar{n}}_{k,\tau }^{m+1}=\frac{1}{\tau }\sum_{k=1}^{\tau }n_{k,\tau }^{m+1}$ and ${\bar{n}}_{k,\tau }^{m}=\frac{1}{\tau }\overset{\tau }{\underset{k=1}{\sum }}n_{k,\tau }^{m}$.

Equation (7) can be written as follows:

(8) $$\begin{array}{c} RCMSE\left(x,\tau ,m,r\right)=-\ln\left(\frac{{\bar{n}}_{k,\tau }^{m+1}}{{\bar{n}}_{k,\tau }^{m}}\right)-\ln\left(\frac{\frac{1}{\tau }\sum_{k=1}^{\tau }n_{k,\tau }^{m+1}}{\frac{1}{\tau }\sum_{k=1}^{\tau }n_{k,\tau }^{m}}\right)\\ =-\ln\left(\frac{\sum_{k=1}^{\tau }n_{k,\tau }^{m+1}}{\sum_{k=1}^{\tau }n_{k,\tau }^{m}}\right) \end{array}$$

#### 2.3.3. Complexity Index (CI)

**Figure 4:**
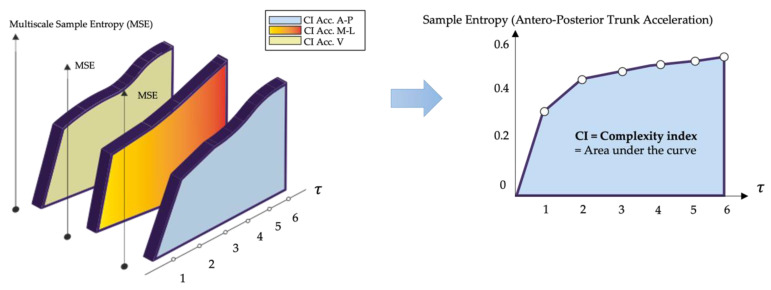
Complexity Index. Multiscale Entropy plot of the triaxial trunk acceleration signal evaluated for all scale factors *τ*; the complexity index was determined for the Antero-Posterior direction of a healthy subject by calculating the area under the curve given by the multiscale Entropy values.

We also use the trapezoid rule to calculate CI by integrating the entropy values over a pre-determined range of scales [56]. This index reflects the amount of information, or entropy, in a signal over a range of time scales, as shown in Figure 4. High entropy values over a wide time scale range, and thus a high CI, indicate high complexity, and vice versa [90]. The maximum scale that can be analyzed depends on the length of the original time series [91].

### 2.4. Clinical Assessment

The HY disease staging system and the motor examination section of the Unified Parkinson’s Disease Rating Scale (UPDRS-III) were used to determine the severity of Parkinson’s disease [92] (Table 1). Clinical scales were administered by an assessor who was not aware of the gait reports. SwPD were classified as fallers (at least one fall) or non-fallers based on a self-reported history of falls in the 6 months preceding the gait assessment [93,94]. A fall was defined as an unintentional landing to the ground that was not caused by a significant intrinsic event or a dangerous situation [93,95]. 

### 2.5. Statistical Analysis

To identify entropy measures with good ability to discriminate between swPD and HS, as represented by an area under the receiver operating characteristics curve (AUC) ≥ 0.70 at a 95% significance level and 80% power under the null hypothesis of an AUC = 0.50, a minimum sample of 68 participants (34 swPD and 34 HS) was calculated. 

After checking the normality of the distributions and equality of the variances through the Shapiro–Wilk and Levene’s test, respectively, a Mann–Whitney test was performed to identify significant differences between swPD and HS in entropy measures at each *τ*, spatio-temporal gait features, pelvic kinematics, HRs, and CV. Cohen’s d with Hedge’s correction were calculated to assess the effect size. 

To identify the entropy measures that best discriminated between swPD and HS, AUCs at each *τ* were calculated. AUCs ≥ 0.70 were deemed to have sufficient overall discriminative ability [96]. To identify the optimal cutoff points (OCPs) for each *τ*, the maximal sum of sensitivity and specificity, Youden Index, and maximal F1 score were calculated. To investigate the likelihood of being correctly classified by a given combination of entropy measure and *τ* at the OCP, positive and negative likelihood ratios (LR+ and LR−, respectively) were calculated and transformed into positive and negative post-test probabilities (PTP+ and PTP−, respectively) through a Fagan’s nomogram [97]. PTPs were also calculated using the prevalence of subjects with gait disorders (35%) [98] in the general older population as pre-test probability to improve the external validity of the results in terms of recognizing gait disorders attributable to PD in aged populations. Diagnostic odds ratios (DORs) were also calculated to assess the diagnostic performances [99]. When F1 score and Youden index score referred to different entropy values, the OCP was chosen as the value with the greatest difference between PTP+ and PTP− [100] (Supplementary Material, Table S2). The combinations of entropy measures and *τ* with the highest DOR, PTPs, the difference between PTP+ and PTP− were considered as the best entropy measures to characterize gait complexity in swPD.

The ability of the identified entropy measures to discriminate between fallers and non-fallers was assessed through a Mann–Whitney test. 

To investigate the ability of the identified entropy measures to discriminate across the disability levels, Kruskal-Wallis test with Dunn’s post hoc analysis and Holm’s correction was performed using the HY stage and the UPDRS III thresholds as between-subjects factors. UPDRS III scores < 32, ≥32, and ≥58 were considered as reflecting mild, moderate, and severe motor disease, respectively [101]. 

To assess the correlations between the identified entropy measures and the clinical features of swPD, spatio-temporal gait characteristics, pelvic kinematics, and the other trunk-acceleration-derived gait indexes that characterize swPD [11,13], a partial correlation analysis excluding the effects of age and gait speed was conducted. 

Statistical analyses were carried out using the IBM SPSS ver. 27, NCSS 2022, and JASP vers. 0.16 software.

## 3. Results

**Figure 5:**
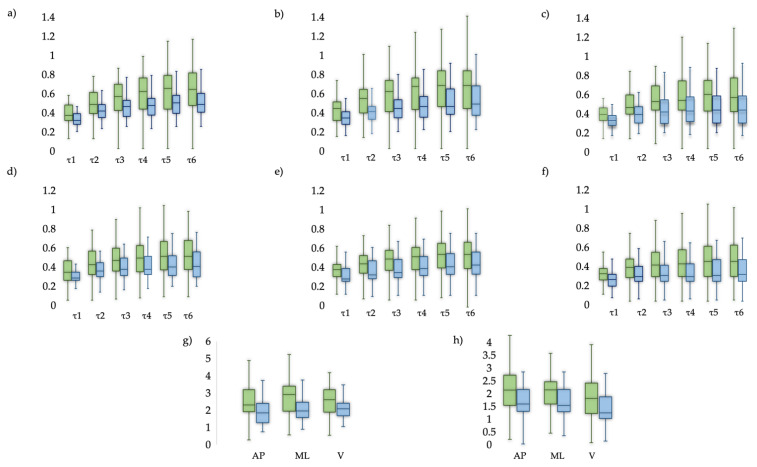
Descriptive measures. Comparison of Entropy metrics between swPD (green) and HS (blue). Boxplots for (**a**) MSE_AP,_ (**b**) MSE_ML,_ (**c**) MSE_V,_ (**d**) RCMSE_AP,_ (**e**) RCMSE_ML_, (**f**) RCMSE_V_, (**g**) CI_MSE,_ (**h**) CI_RCMSE_.

Significant differences between swPD and HS were found in all combinations of entropy measures and *τ* (Figure 5, Supplementary Material: Table S1), and in stride length, pelvic obliquity, pelvic rotation, HRs and CV (Table 1), regardless of age and gait speed. 

**Figure 6:**
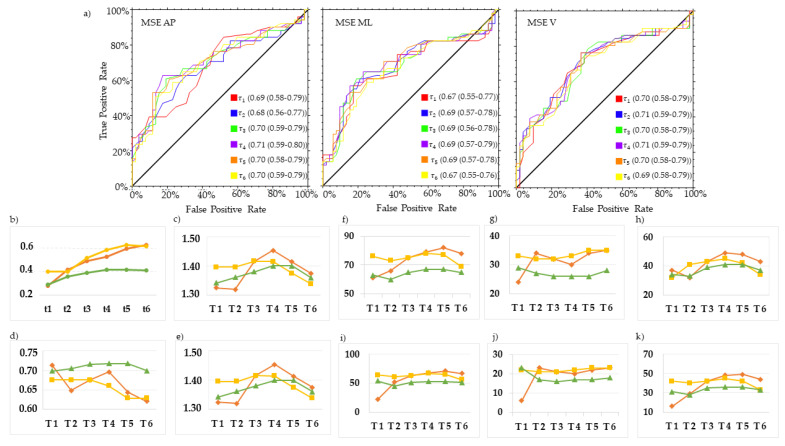
ROC curves and optimal cutoff metrics of MSE. ROC curves of MSE in the antero-posterior (AP) (left), medio-lateral (ML) (middle), and vertical (V) (right) directions of the acceleration signals at *τ*. 1–6. Area under the curves values and their 95% confidence intervals are reported for each *τ* (**a**). Estimated cutoff values (**b**), sum of sensitivity and specificity (**c**), F1 scores (**d**), Youden indexes (**e**), positive post-test probabilities, negative post-test probabilities, and difference between post-test probabilities using the prevalence of PD in the actual sample as pre-test probability (**f**,**g**,**h**, respectively), and using the general 35% prevalence of gait disorders (**i**,**j**,**k**, respectively) are reported. Red lines represent AP direction, yellow lines ML direction, green line V direction.

**Figure 7:**
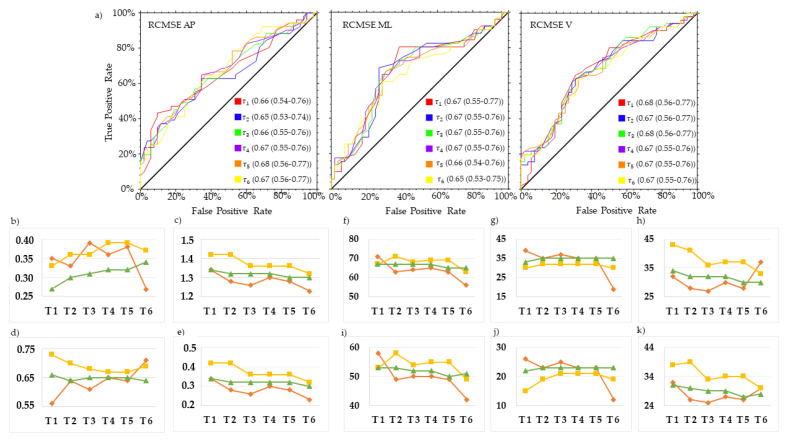
ROC curves and optimal cutoff metrics of RCMSE. ROC curves of RCMSE in the antero-posterior (AP) (left), medio-lateral (ML) (middle), and vertical (V) (right) directions of the acceleration signals at *τ*. 1–6. Area under the curves values and their 95% confidence intervals are reported for each *τ* (**a**). Estimated cutoff values (**b**), sum of sensitivity and specificity (**c**), F1 scores (**d**), Youden indexes (**e**), positive post-test probabilities, negative post-test probabilities, and difference between post-test probabilities using the prevalence of PD in the actual sample as pre-test probability (**f**,**g**,**h**, respectively), and using the general 35% prevalence of gait disorders (**i**,**j**,**k**, respectively) are reported. Red lines represent AP direction, yellow lines ML direction, green line V direction.

**Figure 8:**
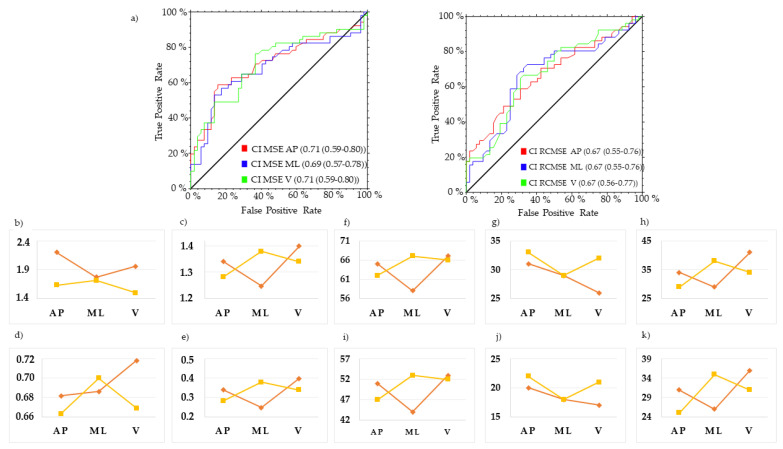
ROC curves and optimal cutoff metrics of CI MSE and CI RCMSE. ROC curves of CI MSE in the antero-posterior (AP) (left), and CI RCMSE (right)medio-lateral (ML). Area under the curves values and their 95% confidence intervals are reported (**a**). Estimated cutoff values (**b**), sum of sensitivity and specificity (**c**), F1 scores (**d**), Youden indexes (**e**), positive post-test probabilities, negative post-test probabilities, and difference between post-test probabilities using the prevalence of PD in the actual sample as pre-test probability (**f**,**g**,**h**, respectively), and using the general 35% prevalence of gait disorders (**i**,**j**,**k**, respectively) are reported. Red lines represent CI MSE, yellow lines represent CI RCMSE.

**Figure 9:**
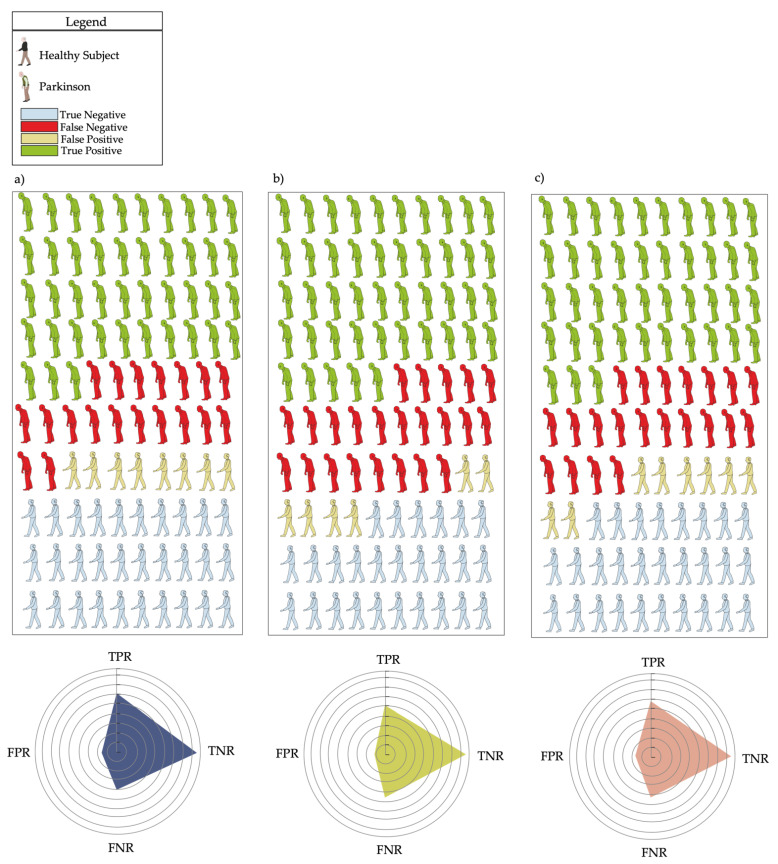
Confusion matrices. Confusion matrices of multiscale entropy in the AP direction at *τ*4 (**a**), and *τ*5 (**b**), and in the medio-lateral direction at *τ4* (**c**). Radar plots represent the true positive (TPR), true negative (TNR), false positive (FPR) and false negative (FNR) rates at each optimal cutoff point.

MSE in the AP direction at *τ*4 (MSE_AP_ *τ*4) and *τ*5 (MSE_AP_ *τ*5), and MSE in the ML direction at *τ*4 (MSE_ML_ *τ*4), revealed the best trade-off between PTP+ and PTP to characterize the gait of swPD, compared with HS (Figure 6, Figure 7 and Figure 8, Supplementary Material: Table S2). Particularly, MSE_AP_ *τ*4 values ≥ 0.53, MSE_AP_ *τ*5 values ≥ 0.60, and MSE_ML_ *τ*4 values ≥ 0.59 characterized swPD with 79%, 82%, and 78%, PTP+, and 30%, 34%, and 33% PTP−, respectively, and the highest DORs (Figure 6, Figure 7, Figure 8 and Figure 9, Supplementary Material: Table S2). After adjusting pre-test probabilities based on the 35% prevalence of gait disorders in elderlies, MSE_AP_ *τ*4, MSE_AP_ *τ*5, and MSE_ML_ *τ*4 still showed the highest differences between PTP+ and PTP− (Figure 6, Supplementary Material: Table S2).

No differences between swPD fallers and non-fallers in MSE_AP_ *τ*4 (*p* = 0.281), MSE_AP_ *τ*5 (*p* = 0.377), and MSE_ML_ *τ*4 (*p* = 0.966) were found.

**Figure 10:**
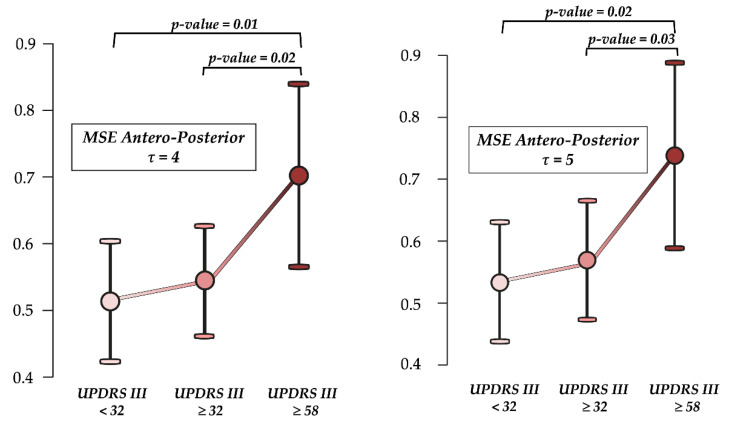
Ability to differentiate across the motor disability levels. Differences in multiscale entropy (MSE) at scale factor *τ*4 and *τ*5 in the antero-posterior direction according to motor disability as assessed by the motor section Unified Parkinson’s Disease Rating Scale (UPDRS III). *p*-values represent significant differences at Dunn’s post-hoc analysis with Holm’s correction after the Kruskal—Wallis’s procedure.

MSE_AP_ *τ*4 (H_2_ = 7.07, *p* = 0.03) and MSE_AP_ *τ*5 (H_2_ = 6.50, *p* = 0.04) differentiated between swPD according to UPDRS III. Post-hoc analysis revealed significant differences in MSE_AP_ *τ*4 and MSE_AP_ *τ*5 between mildly and moderately impaired, and severely impaired swPD (Figure 10). MSE_ML_ *τ*4 did not differentiate across UPDRS III scores (H_2_ = 3.69, *p* = 0.16). No significant differences in age (H_2_ = 1.20, *p* = 0.55) and gait speed (H_2_ = 0.04, *p* = 0.98) were found across the UPDRS III thresholds. No differences across the HY stages in MSE_AP_ *τ*4 (H_2_ = 0.090, *p* = 0.956), MSE_AP_ *τ*5 (H_2_ = 0.105, *p* = 0.949), and MSE_ML_ *τ*4 (H_2_ = 0.357, *p* = 0.836) were found. 

**Figure 11:**
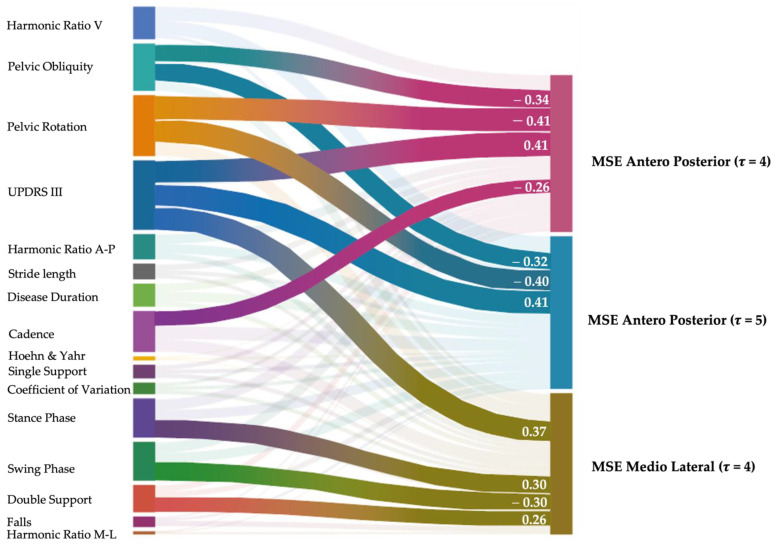
Partial correlation analysis. Partial Spearman’s correlation analysis excluding the effects of age and gait speed between clinical features, spatio-temporal, and kinematic gait characteristics, trunk acceleration derived harmonic ratios, stride length coefficient of variation, and multiscale entropy measures. Highlighted connecting lines represent significant correlations, and their width represents the strength of the correlation, which is also reported numerically. Link: https://public.flourish.studio/visualisation/12786560/ accessed on 15 May 2023).

Regardless of age and gait speed, MSE_AP_ *τ*4, MSE_AP_ *τ*5, and MSE_ML_ *τ*4 positively correlated with UPDRS III. MSE_AP_ *τ*4 and MSE_AP_ *τ*5 negatively correlated with pelvic obliquity and pelvic rotation. MSE_AP_ *τ*4 negatively correlated with cadence. MSE_ML_ *τ*4 positively correlated with the stance and double support phases, and negatively correlated with the swing phase (Figure 11). 

## 4. Discussion

The main objective of this study was to assess the ability of trunk acceleration derived MSE, RCMSE, and CI to characterize swPD gait variability as an expression of the complexity of trunk acceleration signals calculated across a range of *τ* 1–6, regardless of age and gait speed. 

We found that swPD showed higher entropy values than age and gait speed matched HS for all the tested scale factors, and that MSE in the AP direction at *τ*4 and *τ*5, and MSE in the ML direction at *τ*4, provided the best compromise between the probability to identify a subject with PD for values higher than the OCP and the probability to identify an HS for values lower than the OCP. These findings are consistent with previous research, which reported higher entropy values in swPD, indicating lower gait regularity than HS [48,55], and a disruption of trunk accelerations [13] due to the greater number of adjustments required to overcome the increasing instability caused by impaired sensorimotor integration [38]. Conversely, a previous study reported lower entropy values in swPD than healthy controls [102]. Aside from a different method of entropy calculation, this contradictory result may be explained primarily by differences in the healthy control group, which was significantly younger and walked faster than swPD in Kamath’s study compared to our sample. Gait entropy measures are strongly related to age, with younger people exhibiting greater complexity than older people [103,104]. To avoid misrepresenting differences in gait complexity through entropy measures, the ages of the compared groups should be comparable. In this way, because we matched swPD and HS based on age in this study, we reported differences between the groups that are not dependent on age. Furthermore, nonlinear gait indexes are correlated with gait speed [41,58,105], which is known to be reduced and affects most of the spatio-temporal and kinematic gait parameters, potentially overrepresenting the differences between neurotypical and pathological gait [69]. Although we calculated entropy measures directly from trunk acceleration patterns, avoiding the need for step detection, which is a controversial issue in MIMUs- based gait analysis of subjects with neurological conditions [32], we also matched swPD and HS for gait speed. Therefore, our findings allow us to consider MSE in the antero-posterior and medio-lateral directions as age and speed-independent biomarker of gait complexity in swPD. 

In this study, MSE in the AP direction as calculated at *τ*4 and *τ*5, and MSE in the ML direction at *τ*4, outperformed the other scaling configurations in terms of discriminative ability. Riva et al., previously found that *τ*2 represented the best scale factor to identify clinically meaningful gait irregularity through trunk acceleration-derived MSE in older adults [31]. In this way, our findings suggest that higher scaling factors are required to highlight gait irregularities that are caused by Parkinson’s disease rather than aging. In our study, however, MSE_AP_ *τ*4, MSE_AP_ *τ*5, and MSE_ML_ *τ*4 were unable to distinguish between fallers and non-fallers. This finding represents yet another distinction in the calculation of MSE between healthy older adults, where MSE is higher in fallers, and swPD, where the increase in gait irregularity appears to be a direct expression of the clinical features, regardless of fall history. Indeed, we found that MSE values correlated with motor disability, as assessed by UPDRS III, and that MSE in the AP direction was significantly higher in subjects with greater motor impairment. However, we found no differences in entropy values across disease stages as calculated by HY, confirming that gait irregularity in swPD is most likely due to motor symptoms, rather than the longitudinal progression of the disease [49,106], as further reinforced by the lack of correlation with disease duration. Moreover, we found that higher MSE values in the AP direction correlated with lower ranges of movement of the pelvis in the frontal and transverse plane, regardless of age and gait speed. Pelvic rigidity and trunk rotation reduction have been consistently described as characterizing features of swPD [12,17,19,107]. As we directly calculate entropy measures from lower trunk acceleration, we can argue that abnormalities in MSE in the AP direction reflect the irregularity of trunk behavior in swPD due to pelvic rigidity, as an expression of the disruption of trunk acceleration patterns [108]. MSE in the ML direction correlated with stance, swing, and double support phases, which are temporal gait parameters that reflect gait stability in swPD [109,110,111]. In this way, we might hypothesize that MSE_ML_ *τ*4 represents a marker of inefficiency of the compensatory strategy to antero-posterior irregularity [58], resulting in increased medio lateral irregularity. However, because no significant differences were found in temporal gait features between swPD and HS at matched gait speed (Table 1), we cannot ascertain that this mechanism is characteristic of swPD rather than a consequence of the reduced gait speed. As a result, MSE_AP_ *τ*4, MSE_AP_ *τ*5, and MSE_ML_ *τ*4, characterize the irregularity of trunk accelerations during gait, and correlate with the motor symptoms of swPD and reduced pelvic kinematics. The lack of correlation with other trunk acceleration-derived gait indexes that have previously been shown to characterize the gait abnormalities of swPD [13], such as HR and CV, supports the hypothesis of entropy as a measure of gait irregularity that reflects a different aspect of gait variability than the CV [32]. However, because of the relatively high false positive rates (Figure 9), MSE_AP_ *τ*4, MSE_AP_ *τ*5, and MSE_ML_ *τ*4, while providing insights into the gait behavior of swPD, cannot be considered as gait biomarkers alone, requiring additional research into the integration with other gait and clinical features. 

To our knowledge, this is the first application of RCMSE on trunk acceleration derived gait data from swPD. Although significant differences between swPD and HS were found in RCMSE at all scale factors, none of them achieved sufficient discriminative ability to be considered accurate biomarkers of gait irregularity in swPD in this study. Refined algorithms are used on data series with high frequency oscillations. In the field of gait analysis, RCMSE appears to fit better with less predictable signals [89,112], such as electromyographic, than with pre-filtered trunk acceleration patterns at natural steady-state locomotion, which are rather regular and repetitive in time and amplitudes. Analyzing more unstable gait conditions in swPD, such as gait initiation, freezing, and real-world data, could provide additional insights into RCMSE. In this way, MSE was sufficient for the signal typology that we examined. 

In this study, we also assessed CIs. For RCMSE, although significant differences between swPD and HS were found, their discriminative ability was not sufficient to be considered as markers of gait irregularity in swPD. Previous studies have reported increased CI in swPD after rehabilitation [62] or deep brain stimulation [63], indicating that the increase in complexity represents improvements in ability to overcome obstacles during gait [62]. In contrast, Ahmadi et al. reported higher CI values during the over imposed dual task gait condition when compared to natural locomotion [64]. Given the differences in sensor localization and the lack of healthy control groups in the aforementioned studies, a comparison with our results is difficult. In this study, we discovered that lower scale factors, regardless of age or gait speed, were unable to characterize swPD when compared to HS. As a result, the inclusion of non-discriminant entropy values in the CI calculation may have resulted in an underrepresentation of gait irregularity in swPD.

This study presents several limitations. First, in this study we fixed length of 2000 data points, *m* = 2 and *r* = 0.2 times the standard deviation because these parameters are the most used to calculate entropy measures in gait samples. Therefore, our results can be only interpreted based on the aforementioned parameters. To test the relative consistency of our calculations, different combinations of m and r should be tested [43,113]. Furthermore, we analyzed gait data from 30 m walking bouts, which, despite reflecting long time-series in ambulatory settings, limited us to only 2000 useful datapoints in the entropy calculations. Using longer time series from real-world data would allow for more datapoints per scaling factor, likely reflecting more in-depth changes in motor control mechanisms [49,91]. Another limitation of this study is the retrospective self-reported history of falls, which could have led to recall bias. Furthermore, we only assessed swPD during the ON phase of the medication. As differences in entropy measures as measured by shank-mounted MIMUs between ON and OFF phases have been reported in swPD, further studies investigating the ability of trunk acceleration-derived MSE indices to assess the effectiveness of medications are needed. 

## Figures and Tables

**Table 1:** Clinical and spatio-temporal gait characteristics of the included subjects.

		swPD	HS	*p*
Age [mean(SD)]		71.15 (5.12)	69.14 (4.80)	0.06
Gender [n (%)]	F	15 (29.41)	27 (54)	0.01
	M	36 (70.58)	23 (46)	
Disease duration [mean(SD)]		8.04 (4.70)		
HY [n (%)]	1	10 (19.60)		
	2	17 (33.33)		
	3	24 (47.05)		
UPDRS III [mean(SD)]		41.41 (18.22)		
UPDRS III < 32 [n (%)]		16 (31.27)		
UPDRS III ≥ 32 [n (%)]		22 (43.13)		
UPDRS III ≥ 58 [n (%)]		13 (25.49)		
History of falls (n° of falls in the previous 6 months) [mean (SD)]		1.35 (3.28)		
Gait speed (m/s) [mean (SD)]		1.08 (0.25)	1.09 (0.25)	0.91
Stance phase (% gait cycle) [mean (SD)]		60.82 (2.27)	61.41 (3.42)	0.31
Swing phase (% gait cycle) [mean (SD)]		39.18 (2.27)	38.59 (3.42)	0.31
Single support (% gait cycle) [mean (SD)]		39.24 (2.92)	37.93 (5.29)	0.13
Double support (% gait cycle)		10.88 (2.33)	11.90 (4.92)	0.19
Cadence (steps/min) [mean (SD)]		103.37 (20.44)	101.35 (14.06)	0.60
Stride length (m) [mean (SD)]		0.94 (0.21)	1.22 (0.22)	<0.00
Pelvic tilt (°) [mean (SD)]		3.33 (1.55)	3.01 (1.13)	0.25
Pelvic obliquity (°) [mean (SD)]		3.87 (2.16)	5.38 (2.70)	0.01
Pelvic rotation (°) [mean (SD)]		5.49 (3.29)	6.68 (3.90)	0.02
HR AP [mean (SD)]		1.66 (0.26)	2.32 (0.64)	<0.00
HR ML [mean (SD)]		1.62 (0.25)	2.23 (0.59)	<0.00
HR V [mean (SD)]		1.68 (0.28)	2.41 (0.76)	<0.00
stride length CV % [mean (SD)]		39.26 (19.44)	26.69 (13.76)	0.00

swPD, subjects with Parkinson’s disease; HS, age and speed-matched healthy subjects; *p*, significance level at 95% confidence interval in Mann–Whitney procedure; HY, Hoehn and Yahr disease stage classification; UPDRS III, motor section of the Unified Parkinson’s Disease Rating Scale; HR, Harmonic Ratio; AP, antero-posterior direction of the acceleration signal; ML medio-lateral direction of the acceleration signal; V vertical direction of the acceleration signal; CV, coefficient of variation.

## Data Availability

The data presented in this study are available on request from the corresponding author and stored in a password-protected PC located in the Department of Surgical Sciences and Biotechnologies, University of Rome Sapienza.

## Supplementary Materials

The following supporting information can be downloaded at: https://www.mdpi.com/article/10.3390/s23104983/s1, Table S1: Differences in Entropy measures between swPD and HS. Table S2: Discriminative ability of the entropy measures.
