# Supplementary material for: Multiscale Entropy Algorithms to Analyze Complexity and Variability of Trunk Accelerations Time Series in Subjects with Parkinson’s Disease
Source: Sensors (Basel). 2023 May 22;23(10):4983. doi: 10.3390/s23104983 (PMC10221101; doi:10.3390/s23104983)
Supplement: Supplementary file 1 [file sensors-23-04983-s001.zip › sensors-2317533-supplementary.pdf]

**SUPPLEMENTARY MATERIAL**

Table S1. Differences in entropy measures between swPD and HS.

| Entropy measure | Direction | τ | swPD [mean (SD)] | HS [mean (SD)] | p | Cohen's d |
|---|---|---|---|---|---|---|
| MSE | AP | τ 1 | 0.35 (0.10) | 0.30 (0.06) | 0.00 | 0.66 |
| | | τ 2 | 0.45 (0.15) | 0.38 (0.09) | 0.00 | 0.61 |
| | | τ 3 | 0.52 (0.17) | 0.42 (0.12) | < 0.00 | 0.68 |
| | | τ 4 | 0.56 (0.20) | 0.44 (0.14) | < 0.00 | 0.74 |
| | | τ 5 | 0.59 (0.22) | 0.45 (0.15) | < 0.00 | 0.71 |
| | | τ 6 | 0.60 (0.23) | 0.46 (0.16) | < 0.00 | 0.72 |
| | ML | τ 1 | 0.38 (0.14) | 0.32 (0.09) | 0.01 | 0.56 |
| | | τ 2 | 0.48 (0.20) | 0.38 (0.12) | 0.00 | 0.62 |
| | | τ 3 | 0.55 (0.25) | 0.42 (0.15) | 0.00 | 0.64 |
| | | τ 4 | 0.60 (0.29) | 0.44 (0.17) | 0.00 | 0.66 |
| | | τ 5 | 0.64 (0.33) | 0.46 (0.18) | 0.00 | 0.66 |
| | | τ 6 | 0.65 (0.34) | 0.48 (0.20) | 0.01 | 0.59 |
| | V | τ 1 | 0.33 (0.10) | 0.28 (0.09) | 0.00 | 0.54 |
| | | τ 2 | 0.43 (0.15) | 0.34 (0.12) | < 0.00 | 0.66 |
| | | τ 3 | 0.49 (0.19) | 0.38 (0.14) | < 0.00 | 0.68 |
| | | τ 4 | 0.52 (0.22) | 0.38 (0.16) | < 0.00 | 0.73 |
| | | τ 5 | 0.54 (0.24) | 0.40 (0.16) | 0.00 | 0.69 |
| | | τ 6 | 0.55 (0.25) | 0.40 (0.17) | 0.00 | 0.70 |
| RCMSE | AP | τ 1 | 0.31 (0.09) | 0.26 (0.07) | 0.01 | 0.63 |
| | | τ 2 | 0.38 (0.12) | 0.32 (0.09) | 0.02 | 0.55 |
| | | τ 3 | 0.42 (0.14) | 0.34 (0.10) | 0.01 | 0.60 |
| | | τ 4 | 0.44 (0.17) | 0.35 (0.11) | 0.00 | 0.64 |
| | | τ 5 | 0.46 (0.18) | 0.36 (0.12) | 0.00 | 0.63 |
| | | τ 6 | 0.47 (0.20) | 0.37 (0.12) | 0.00 | 0.62 |
| | ML | τ 1 | 0.31 (0.11) | 0.27 (0.08) | 0.01 | 0.49 |
| | | τ 2 | 0.38 (0.16) | 0.31 (0.11) | 0.01 | 0.53 |
| | | τ 3 | 0.43 (0.20) | 0.33 (0.13) | 0.01 | 0.53 |
| | | τ 4 | 0.45 (0.24) | 0.35 (0.14) | 0.01 | 0.54 |
| | | τ 5 | 0.48 (0.27) | 0.37 (0.14) | 0.01 | 0.52 |
| | | τ 6 | 0.50 (0.30) | 0.38 (0.15) | 0.02 | 0.50 |
| | V | τ 1 | 0.28 (0.09) | 0.24 (0.08) | 0.00 | 0.52 |
| | | τ 2 | 0.35 (0.13) | 0.28 (0.11) | 0.01 | 0.58 |

| | | | | | |
|---|---|---|---|---|---|
| | τ 3 | 0.38 (0.17) | 0.29 (0.13) | 0.00 | 0.62 |
| | τ 4 | 0.40 (0.19) | 0.30 (0.13) | 0.01 | 0.63 |
| | τ 5 | 0.41 (0.20) | 0.31 (0.14) | 0.01 | 0.61 |
| | τ 6 | 0.42 (0.21) | 0.31 (0.14) | 0.01 | 0.60 |
| CI MSE | AP | 2.59 (0.89) | 2.06 (0.59) | < 0.00 | 0.71 |
| | ML | 2.78 (1.29) | 2.10 (0.75) | 0.00 | 0.64 |
| | V | 2.42 (0.96) | 1.84 (0.69) | < 0.00 | 0.70 |
| CI RCMSE | AP | 2.08 (0.75) | 1.69 (0.49) | 0.01 | 0.63 |
| | ML | 2.14 (1.07) | 1.68 (0.62) | 0.01 | 0.53 |
| | V | 1.90 (0.82) | 1.45 (0.06) | 0.01 | 0.61 |

swPD, subjects with Parkinson's disease; HS, age and gait speed – matched healthy subjects; p, significance level at 95% CI in Mann – Whitney procedure; MSE, multiscale sample entropy; RCMSE, refined composite multiscale entropy; CI, complexity index; AP, antero-posterior direction of the acceleration signal; ML, medio-lateral direction of the acceleration signal; V, vertical direction of the acceleration signal.

Table S2. Discriminative ability of the entropy measures

| Entropy measure | Tau | AUC (95% CI) | OCP | F1 score | Se+SP | Youden index | DOR | LR+ | LR- | PTP+ | PTP- | Δ PTP | PTP+ *adj* | PTP- *adj* | Δ PTP *adj* | |
|---|---|---|---|---|---|---|---|---|---|---|---|---|---|---|---|---|
| MSE AP | t1 | 0.69 (0.58 - 0.79) | ≥ 0.28 | 0.71 | 1.32 | 0.32 | 4.98 | 1.62 | 0.33 | 61 | 24 | 37 | 22 | 6 | 16 | * |
| | t2 | 0.68 (0.56 - 0.77) | ≥ 0.34 | 0.69 | 1.27 | 0.27 | 3.70 | 1.48 | 0.40 | 59 | 27 | 32 | 21 | 7 | 14 | |
| | | | ≥ 0.42 | 0.65 | 1.32 | 0.32 | 3.78 | 2.04 | 0.54 | 66 | 34 | 32 | 52 | 23 | 29 | * |
| | t3 | 0.70 (0.59 - 0.79) | ≥ 0.49 | 0.68 | 1.42 | 0.42 | 6.58 | 3.16 | 0.48 | 75 | 32 | 43 | 63 | 21 | 42 | * |
| | t4 | 0.71 (0.59 -0.80) | ≥ 0.53 | 0.70 | 1.45 | 0.45 | 8.73 | 3.93 | 0.45 | 79 | 30 | **49** | 68 | 20 | **48** | * |
| | t5 | 0.70 (0.58 - 0.79) | ≥ 0.45 | 0.68 | 1.27 | 0.27 | 3.25 | 1.53 | 0.47 | 59 | 31 | 28 | 45 | 20 | 25 | |
| | | | ≥ 0.60 | 0.64 | 1.41 | 0.41 | 8.66 | 4.59 | 0.53 | 82 | 34 | **48** | 71 | 22 | **49** | * |
| | t6 | 0.70 (0.59 - 0.79) | ≥ 0.43 | 0.69 | 1.29 | 0.29 | 3.80 | 1.55 | 0.41 | 60 | 28 | 32 | 45 | 18 | 27 | |
| | | | ≥ 0.63 | 0.62 | 1.38 | 0.38 | 6.63 | 3.78 | 0.57 | 78 | 35 | 43 | 67 | 23 | 44 | * |
| MSE ML | t 1 | 0.67 (0.55 - 0.77) | ≥ 0.32 | 0.69 | 1.32 | 0.32 | 3.99 | 1.76 | 0.44 | 63 | 30 | 33 | 49 | 19 | 30 | |
| | | | ≥ 0.4 | 0.65 | 1.40 | 0.40 | 6.31 | 3.28 | 0.52 | 76 | 33 | 43 | 64 | 22 | 42 | * |
| | t 2 | 0.69 (0.57 - 0.78) | ≥ 0.35 | 0.69 | 1.27 | 0.27 | 3.51 | 1.49 | 0.43 | 59 | 29 | 30 | 45 | 19 | 26 | |
| | | | ≥ 0.40 | 0.68 | 1.40 | 0.40 | 5.86 | 2.87 | 0.49 | 73 | 32 | 41 | 61 | 21 | 40 | * |
| | t3 | 0.69 (0.56 - 0.78) | ≥ 0.35 | 0.68 | 1.23 | 0.23 | 3.49 | 1.98 | 0.57 | 65 | 35 | 30 | 52 | 23 | 29 | |
| | | | ≥ 0.52 | 0.68 | 1.42 | 0.42 | 6.58 | 3.16 | 0.48 | 75 | 32 | 43 | 63 | 21 | 42 | * |
| | t 4 | 0.69 (0.57 - 0.79) | ≥ 0.53 | 0.69 | 1.42 | 0.42 | 6.11 | 2.80 | 0.46 | 73 | 30 | 43 | 60 | 20 | 40 | |
| | | | ≥ 0.59 | 0.66 | 1.42 | 0.42 | 7.24 | 3.69 | 0.51 | 78 | 33 | **45** | 67 | 22 | **45** | * |
| | t 5 | 0.69 (0.57 - 0.78) | ≥ 0.43 | 0.69 | 1.30 | 0.30 | 3.93 | 1.63 | 0.42 | 61 | 28 | 33 | 47 | 18 | 29 | |
| | | | ≥ 0.63 | 0.63 | 1.38 | 0.38 | 6.25 | 3.44 | 0.55 | 77 | 35 | 42 | 65 | 23 | 42 | * |
| | t 6 | 0.67 (0.55 - 0.76) | ≥ 0.37 | 0.67 | 1.19 | 0.19 | 2.69 | 1.30 | 0.48 | 55 | 32 | 23 | 41 | 21 | 20 | |
| | | | ≥ 0.62 | 0.64 | 1.34 | 0.34 | 4.27 | 2.35 | 0.55 | 69 | 35 | 34 | 56 | 23 | 33 | * |
| MSE V | t 1 | 0.70 (0.58 - 0.79) | ≥ 0.29 | 0.70 | 1.34 | 0.34 | 4.43 | 1.81 | 0.41 | 63 | 29 | 34 | 49 | 18 | 31 | * |
| | | | ≥ 0.33 | 0.63 | 1.32 | 0.32 | 3.89 | 2.18 | 0.56 | 68 | 35 | 33 | 54 | 23 | 31 | |
| | t 2 | 0.71 (0.59 - 0.79) | ≥ 0.36 | 0.71 | 1.36 | 0.36 | 4.00 | 1.53 | 0.38 | 60 | 27 | 33 | 45 | 17 | 28 | * |
| | t 3 | 0.70 (0.58 - 0.79) | ≥ 0.39 | 0.72 | 1.38 | 0.38 | 5.37 | 1.94 | 0.36 | 65 | 26 | 39 | 51 | 16 | 35 | * |
| | t 4 | 0.71 (0.59 - 0.79) | ≥ 0.42 | 0.72 | 1.40 | 0.40 | 5.65 | 2.09 | 0.37 | 67 | 26 | 41 | 53 | 17 | 36 | * |
| | t 5 | 0.70 (0.58 - 0.79) | ≥ 0.42 | 0.72 | 1.40 | 0.40 | 5.65 | 2.09 | 0.37 | 67 | 26 | 41 | 53 | 17 | 36 | * |
| | t 6 | 0.69 (0.58 - 0.78) | ≥ 0.41 | 0.70 | 1.36 | 0.36 | 4.71 | 1.93 | 0.41 | 65 | 28 | 37 | 51 | 18 | 33 | * |
| RCMSE AP | t 1 | 0.66 (0.54 - 0.76) | ≥ 0.21 | 0.68 | 1.11 | 0.11 | 2.80 | 1.14 | 0.41 | 52 | 28 | 24 | 38 | 18 | 20 | |
| | | | ≥ 0.35 | 0.56 | 1.34 | 0.34 | 3.94 | 2.60 | 0.66 | 71 | 39 | 32 | 58 | 26 | 32 | * |
| | t 2 | 0.65 (0.53 - 0.74) | ≥ 0.16 | 0.68 | 1.06 | 0.06 | 4.17 | 1.06 | 0.26 | 50 | 19 | 31 | 36 | 12 | 24 | |

| | | | | | | | | | | | | | | | | |
|---|---|---|---|---|---|---|---|---|---|---|---|---|---|---|---|---|
| | | | ≥ 0.33 | 0.64 | 1.28 | 0.28 | 3.18 | 1.81 | 0.57 | 63 | 35 | 28 | 49 | 23 | 26 | * |
| | t 3 | 0.66 (0.55 - 0.76) | ≥ 0.16 | 0.68 | 1.06 | 0.06 | 3.06 | 1.04 | 0.34 | 50 | 25 | 25 | 36 | 15 | 21 | |
| | | | ≥ 0.39 | 0.61 | 1.26 | 0.26 | 2.98 | 1.85 | 0.62 | 64 | 37 | 27 | 50 | 25 | 25 | * |
| | t 4 | 0.67 (0.55 - 0.76) | ≥ 0.30 | 0.68 | 1.23 | 0.23 | 3.16 | 1.38 | 0.44 | 57 | 29 | 28 | 43 | 19 | 24 | |
| | | | ≥ 0.36 | 0.65 | 1.30 | 0.30 | 3.46 | 1.87 | 0.54 | 65 | 35 | 30 | 50 | 23 | 27 | * |
| | t 5 | 0.68 (0.56 - 0.77) | ≥ 0.30 | 0.69 | 1.23 | 0.23 | 3.36 | 1.37 | 0.41 | 57 | 28 | 29 | 42 | 18 | 24 | |
| | | | ≥ 0.38 | 0.64 | 1.28 | 0.28 | 3.16 | 1.77 | 0.56 | 63 | 35 | 28 | 49 | 23 | 26 | * |
| | t 6 | 0.67 (0.56 - 0.77) | ≥ 0.27 | 0.71 | 1.23 | 0.23 | 5.22 | 1.33 | 0.26 | 56 | 19 | 37 | 42 | 12 | 30 | * |
| | | | ≥ 0.38 | 0.63 | 1.26 | 0.26 | 2.92 | 1.72 | 0.59 | 63 | 37 | 26 | 48 | 24 | 24 | |
| RCMSE ML | t 1 | 0.67 (0.55 - 0.77) | ≥ 0.26 | 0.73 | 1.42 | 0.42 | 6.53 | 2.09 | 0.32 | 67 | 24 | 43 | 53 | 15 | 38 | * |
| | t 2 | 0.67 (0.55 - 0.76) | ≥ 0.33 | 0.70 | 1.42 | 0.42 | 5.93 | 2.55 | 0.43 | 71 | 30 | 41 | 58 | 19 | 39 | * |
| | | | ≥ 0.30 | 0.70 | 1.32 | 0.32 | 4.43 | 1.67 | 0.38 | 62 | 28 | 34 | 47 | 17 | 30 | |
| | t3 | 0.67 (0.55 - 0.76) | ≥ 0.36 | 0.68 | 1.36 | 0.36 | 4.52 | 2.17 | 0.48 | 68 | 32 | 36 | 54 | 21 | 33 | * |
| | t 4 | 0.67 (0.55 - 0.76) | ≥ 0.32 | 0.70 | 1.30 | 0.30 | 4.10 | 1.61 | 0.39 | 61 | 27 | 34 | 46 | 17 | 29 | |
| | | | ≥ 0.39 | 0.67 | 1.36 | 0.36 | 4.57 | 2.24 | 0.49 | 69 | 32 | 37 | 55 | 21 | 34 | * |
| | t 5 | 0.66 (0.54 - 0.76) | ≥ 0.36 | 0.69 | 1.32 | 0.32 | 3.99 | 1.76 | 0.44 | 63 | 30 | 33 | 49 | 19 | 30 | |
| | | | ≥ 0.39 | 0.67 | 1.36 | 0.36 | 4.57 | 2.24 | 0.49 | 69 | 32 | 37 | 55 | 21 | 34 | * |
| | t 6 | 0.65 (0.53 - 0.75) | ≥ 0.37 | 0.69 | 1.32 | 0.32 | 3.99 | 1.76 | 0.44 | 63 | 30 | 33 | 49 | 19 | 30 | * |
| | | | ≥ 0.42 | 0.64 | 1.32 | 0.32 | 3.84 | 2.11 | 0.55 | 67 | 35 | 32 | 53 | 23 | 30 | |
| RCMSE V | t 1 | 0.68 (0.56 - 0.77) | ≥ 0.23 | 0.70 | 1.30 | 0.30 | 4.10 | 1.61 | 0.39 | 61 | 27 | 34 | 46 | 17 | 29 | |
| | | | ≥ 0.27 | 0.66 | 1.34 | 0.34 | 4.12 | 2.10 | 0.51 | 67 | 33 | 34 | 53 | 22 | 31 | * |
| | t 2 | 0.67 (0.56 - 0.77) | ≥ 0.24 | 0.70 | 1.27 | 0.27 | 3.94 | 1.46 | 0.37 | 58 | 26 | 32 | 44 | 17 | 27 | |
| | | | ≥ 0.30 | 0.64 | 1.32 | 0.32 | 3.82 | 2.10 | 0.55 | 67 | 35 | 32 | 53 | 23 | 30 | * |
| | t 3 | 0.68 (0.56 - 0.77) | ≥ 0.24 | 0.70 | 1.27 | 0.27 | 4.26 | 1.45 | 0.34 | 58 | 25 | 33 | 44 | 15 | 29 | |
| | | | ≥ 0.31 | 0.65 | 1.32 | 0.32 | 3.78 | 2.04 | 0.54 | 67 | 35 | 32 | 52 | 23 | 29 | * |
| | t 4 | 0.67 (0.55 - 0.76) | ≥ 0.21 | 0.68 | 1.13 | 0.13 | 3.15 | 1.17 | 0.37 | 53 | 26 | 27 | 39 | 17 | 22 | |
| | | | ≥ 0.32 | 0.65 | 1.32 | 0.32 | 3.78 | 2.04 | 0.54 | 67 | 35 | 32 | 52 | 23 | 29 | * |
| | t 5 | 0.67 (0.55 - 0.76) | ≥ 0.25 | 0.69 | 1.25 | 0.25 | 3.42 | 1.43 | 0.42 | 58 | 29 | 29 | 44 | 18 | 26 | |
| | | | ≥ 0.32 | 0.65 | 1.30 | 0.30 | 3.46 | 1.87 | 0.54 | 65 | 35 | 30 | 50 | 23 | 27 | * |
| | t 6 | 0.67 (0.55 - 0.76) | ≥ 0.24 | 0.69 | 1.21 | 0.21 | 3.33 | 1.32 | 0.40 | 56 | 28 | 28 | 42 | 18 | 24 | |
| | | | ≥ 0.34 | 0.64 | 1.30 | 0.30 | 3.49 | 1.92 | 0.55 | 65 | 35 | 30 | 51 | 23 | 28 | * |
| CI MSE AP | | 0.71 (0.59 - 0.80) | ≥ 2.20 | 0.68 | 1.34 | 0.34 | 4.17 | 1.93 | 0.46 | 65 | 31 | 34 | 51 | 20 | 31 | * |
| CI MSE ML | | 0.69 (0.57 - 0.78) | ≥ 1.77 | 0.69 | 1.25 | 0.25 | 3.42 | 1.43 | 0.42 | 58 | 29 | 29 | 44 | 18 | 26 | * |
| CI MSE V | | 0.71 (0.59 - 0.80) | ≥ 1.96 | 0.72 | 1.40 | 0.40 | 5.65 | 2.09 | 0.37 | 67 | 26 | 41 | 53 | 17 | 36 | * |
| Ci RCMSE AP | | 0.67 (0.55 - 0.76) | ≥ 1.63 | 0.66 | 1.28 | 0.28 | 3.27 | 1.67 | 0.51 | 62 | 33 | 29 | 47 | 22 | 25 | * |
| CI RCMSE ML | | 0.67 (0.55 - 0.76) | ≥ 1.71 | 0.70 | 1.38 | 0.38 | 4.99 | 2.10 | 0.42 | 67 | 29 | 38 | 53 | 18 | 35 | * |
| CI RCMSE V | | 0.67 (0.56 - 0.77) | ≥ 1.50 | 0.67 | 1.34 | 0.34 | 4.12 | 2.04 | 0.50 | 66 | 32 | 34 | 52 | 21 | 31 | * |

AUC, area under the receiver operating characteristics curve; OCP, optimal cutoff point; Se+Sp, maximal sum of sensitivity and specificity values, DOR, diagnostic odds ratio; LR+, positive likelihood ratio; LR-, negative likelihood ratio; PTP+, positive post-test probability; PTP-, negative post-test probability; PTP adj, post-test probabilities based on the reported 35% prevalence of gait disorders in older adults; MSE, multiscale sample entropy; RCMSE, refined composite multiscale entropy; CI, complexity index; AP, antero-posterior direction of the acceleration signal; ML, medio-lateral direction of the acceleration signal; V, vertical direction of the acceleration signal; *, selected OCPs.
